# Insecticidal effect of aconitine on the rice brown planthoppers

**DOI:** 10.1371/journal.pone.0221090

**Published:** 2019-08-19

**Authors:** Shuqin Wei, Huijuan Zhang, Bo Li, Jianfen Ji, Xiwen Shao

**Affiliations:** 1 College of Agronomy, Jilin Agricultural University, Changchun, China; 2 College of Agronomy, Jilin College of Agricultural Science and Technology, Jilin, Jilin, China; 3 College of Biological and Pharmaceutical Engineering, Jilin College of Agricultural Science and Technology, Jilin, Jilin, China; Zhejiang University, CHINA

## Abstract

The brown planthopper, *Nilaparvata lugens* (Stål), severely damages rice production and develops high level resistance to several classes of insecticides. To find potential insecticidal resources is always important. As an environmentally friendly compound, aconitine exhibits potential pesticide features. In the present study, the pesticide and knockdown effects of aconitine were first tested on the brown planthopper. The results showed that the knockdown rates for an aconitine concentration of 200 ppm was 83.6%. The insecticidal LD_50_ was 22.68 ng/pest (95% CI, 17.75–28.99). The molecular mechanisms responding to aconitine application were analyzed through transcriptional sequencing. Compared to that of the knockdown nymphs of the brown planthoppers, the enzymes CYP3A4, UDP-glucuronosyltransferase (UGT), GST, carboxylesterase (EC3.1.1.1), and GABAergic synapse were up-regulated. We inferred that aconitine might be neurotoxic to the brown planthoppers, and the conscious nymphs resist the drug neurotoxicity through the upregulation of CYP3A4, UGT, and GABA receptor mutation. Although aconitine is not safe for mammals, it may be a leading compound to develop novel insecticides.

## Introduction

The brown planthopper (*Nilaparvata lugens* Stål) feeds mainly on rice plants and often causes large yield loss, and is, therefore, one of the most important insect pests of rice [1]. This insect infests rice crops at all stages of plant growth and damages rice directly through feeding and also by transmitting rice viruses. Up to 60% yield loss caused by the brown planthoppers is common in susceptible rice cultivars. Excessive use of urea as nitrogenous fertilizer and insecticides often leads to outbreaks by increasing the fecundity of brown planthoppers and by reducing populations of natural enemies [2–6].

The brown planthopper often exhibits resistance to chemical insecticides from different classes, including organophosphorus, carbamate, and neonicotinoid insecticides, which urges us to find new potential compounds for novel insecticide development [7–12]. The resistance mechanisms of insects exhibit two patterns, metabolic resistance and target site resistance, as reviewed by Hemingway et al. (2004). Metabolic resistance mechanisms involve carboxylesterases, cytochrome P450-dependent monooxygenases, and glutathione S-transferases (GSTs). Target site resistance mechanisms involve insensitive acetylcholinesterase, GABA receptor mutation, and mutations in the voltage-gated sodium channel.

Resistance to insecticides urges us to seek new potential compounds to be used as insecticides. Plant alkaloids serve as potential leads for novel insecticides [13]. Aconitine exhibits potential pesticide features and good degradability as an environment-friendly compound, thus, there is increasing interest to perform further studies on this compound [14, 15]. However, the effects and molecular mechanisms of aconitine to knockdown insects or provide resistance are still unknown. The current study tested the insecticidal effects of aconitine on brown planthoppers while a transcriptomic analysis following aconitine treatments was performed to determine the molecular mechanisms impacting the survival following the knockdown of insects after exposure to aconitine.

## Materials and methods

### Chemicals

Aconitine (HPLC purity 98.72%) was purchased from Chengdu MUST Biotech Ltd. Co. (Chengdu, China). Ethanol was purchased from Shanghai Lingfeng Chemical Reagent Co., Ltd. (Shanghai, China).

### Insects

The susceptible strain of the brown planthoppers was donated by the Insecticide Pharmacology and Neurotoxicology Laboratory, College of Plant Protection, Nanjing Agricultural University (Nanjing, China). Insects were kept in an insect incubator with rice seedlings at 27 ± 1°C with 70–80% humidity and 16/8 h light/dark photoperiod.

### Bioassay

The bioassay was performed by a topical application method as previously described [16]. Aconitine was dissolved in ethanol and diluted into 50, 100, 200, 400, and 800 mg/L in ethanol. The 4^th^ instar nymphs were anesthetized by CO_2_, and then a droplet (25 nL) of aconitine solution was immediately applied onto the pro thorax notum of each nymph using the UltraMicro Pump (UMP2) Microsyringe Injector (World Precision Instruments, Inc., Sarasota, FL, United States). The bioassay was repeated three times for each concentration, with each replication including 30 nymphs. As the control (CK), 25 nL ethanol was used. After treatment, the nymphs were transferred into the bioassay cup with rice seedlings and medium. Then they were cultured in the insect incubators at 25°C. After 48 h, the mortality of the nymphs was recorded in order to calculate the 50% lethal dose (LD_50_) of aconitine against the brown planthoppers. The treated insects that did not react to a touch with a soft brush were considered dead. To estimate the knockdown effects of aconitine, the dose of 5 ng/pest (200 mg/L, 25 nL) was applied to 4^th^ instar nymphs. The number of conscious insects was counted at certain time intervals from 0.5 to 12 h after the aconitine application.

### Transcriptional sequencing and analysis

For transcriptome sequencing, the 25 nL (200 mg/L) of aconitine and ethanol (as control) were applied to the 4th instar nymphs. After 6 h, the knockdown and active insects following exposure to aconitine, and the active nymphs in the control were sampled, respectively. After 48 h, the surviving insects exposed to aconitine and ethanol were collected. For each sample, 20 nymphs were randomly collected. The total RNA was isolated using the TRIzol reagent (Invitrogen, Carlsbad, CA) following the manufacturer's instructions. The RNA purity and integrity were evaluated by the absorbance ratio at 260 nm and by agarose gel electrophoresis. The qualified RNA was used for cDNA library construction.

Transcriptional sequencing was performed with the Illumina Hiseq2000 platform by the BGI Company (Shenzhen, Guangdong, China). The sequencing reads which containing low-quality, adaptor-polluted and high content of unknown base (N) reads, should be processed to be removed to obtain the clean data. The clean reads were mapped to the reference genome of *N*. *lugens* (GenBank Accession Number: AOSB00000000.1, assembly NilLug1.0) using Bowtie2 (version: v2.2.5), and then predicted the novel transcripts. The novel transcripts that potentially encoding the proteins were added to the references. The gene expression level was calculated by RSEM (RNASeq by Expectation Maximization) and then the different expressed genes (DEGs) were detected between different samples. With the GO annotation result, we classified DEGs according to official classification, and we also performed GO functional enrichment using phyper, a function of R. With the KEGG annotation result, we classified DEGs according to official classification, and we also performed pathway functional enrichment using phyper.

## Results

### The toxicity and knockdown effect of aconitine

A bioassay was performed to establish the LD_50_ of aconitine against the brown planthoppers. A log-dose probit line formula y = 1.7447x + 3.3415 was generated and presented in a good linear manner. The calculated LD_50_ was 8.92 ng/pest (95% CI, 7.4077–10.7523 ng/pest).

**Fig 1:**
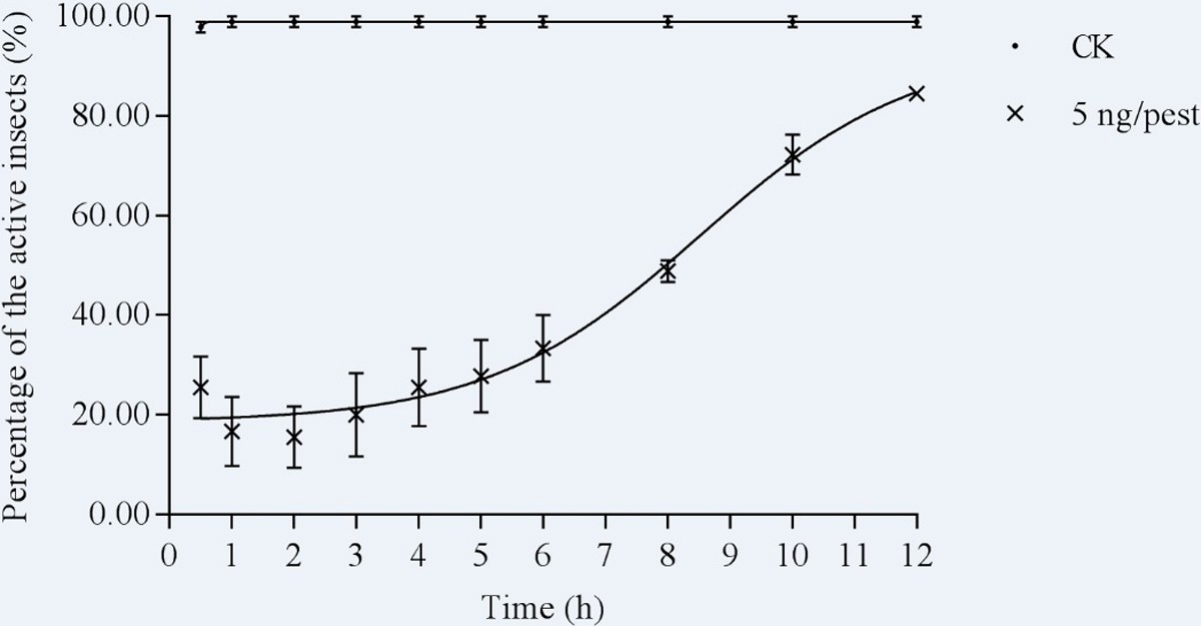
The knockdown effect curve of aconitine against the brown planthopper. Data are mean ± SEM of three replications. CK, 25 nL ethanol as control.

Based on the result of the toxicity bioassay, the dose of 5 ng/pest (the theoretical mortality rate, 22.62%) was applied in the knockdown experiment. The active rate of the aconitine-treated insects was 26% at 0.5 h and decreased to about 15% during the following 2 hours. At 4–6 h after aconitine treatment, the percentage of the active insects increased slightly, and some knockdown nymphs began recovering after 6 h (Fig 1). The active rate reached 83.6% at 12 h after aconitine treatment. In contrast, the active rate of CK was 93% at 0.5 h and then increased to 97% after 1 h, which indicated the knockdown effect was caused by aconitine but not by carbon dioxide(S1 File).

### General analysis of transcriptome data

The Illumina Hiseq2000 sequencing produced the average of 4.29 Gb clean data for each sample. On average, 73.36% and 70.87% of clean reads from each sample were mapped to the genome and gene set, respectively. A total of 15 045 novel transcripts were predicted, in which 7 647 transcripts encoded the alternative splicing of the known proteins, 1 850 transcripts encoded novel proteins, and others were long non-coding RNAs. Additional information about the transcriptome is provided in the supplemental information (S1 Table).

### Differentially expressed genes in the knockdown insects

**Fig 2:**
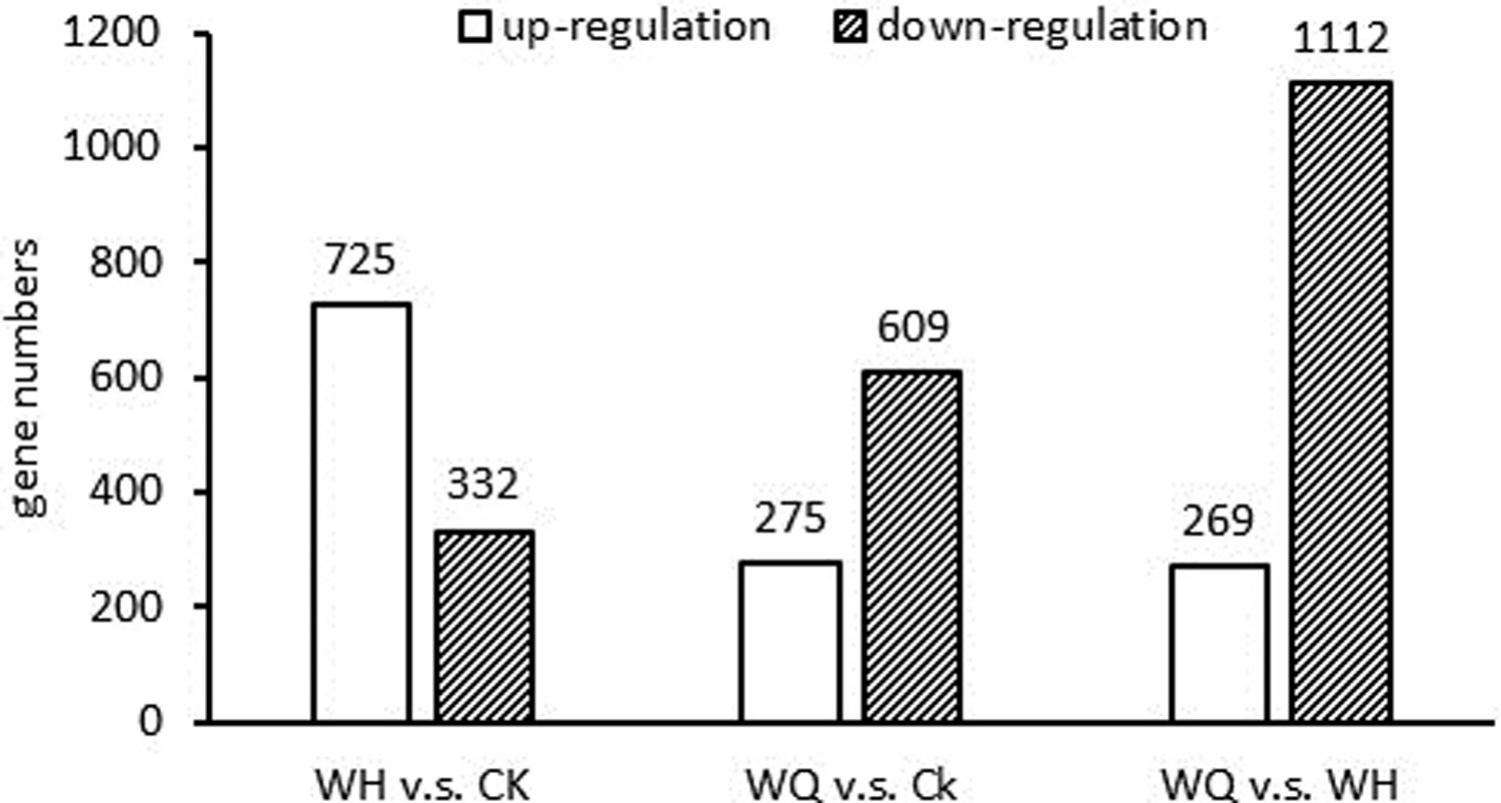
Differential genes among multiple comparison. CK: control; WH: knockdown insects; WQ: active insects.

To investigate the knockdown and recovery mechanism of the brown planthopper treated by aconitine, a comparison of the knockdown insects with the control insects at 6 h was performed. Compared with the control (no aconitine treatment), 1 057 genes were differentially regulated in the knockdown insects, including 725 up-regulated and 332 down-regulated genes. In the active insects, 884 genes were differentially regulated, representing 275 up-regulated and 609 down-regulated genes. In the comparison of the active and knockdown insects, 269 genes were up-regulated (Fig 2).

**Fig 3:**
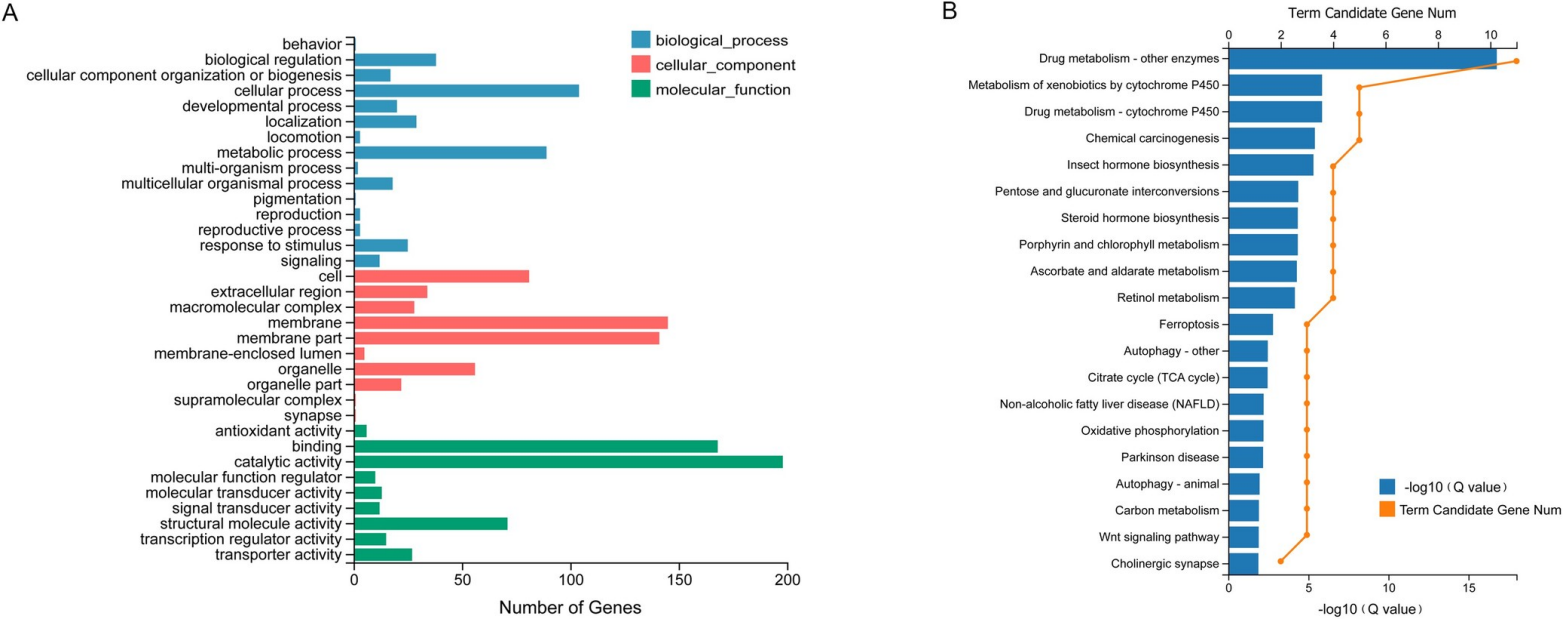
GO enrichment of differential genes compared between knockdown insects and control at 6 h. (A) GO enrichment of differentially expressed genes. (B) The KEGG pathway enrichment of differentially expressed genes in the xenobiotics biodegradation and metabolism term. Rich Ratio = Term Candidate Gene Num / Term Gene Num.

All differentially expressed genes were enriched into 20 GO terms, among which the catalytic activity (198 genes) and binding (168 genes) were much higher than most other terms (Fig 3A). Furthermore, the xenobiotics biodegradation and metabolism pathways were enriched in the drug metabolism by P450s and other enzymes (Fig 3B). CYP3A4 and glucuronosyltransferase (UDPGT, UGT) enzymes, which are involved in irinotecan metabolism, were down-regulated indicating a decrease in detoxication ability of the knockdown nymphs, making the insects sensitive to xenobiotics (S1 and S2 Figs). These results were consistent with the knockdown effects.

**Fig 4:**
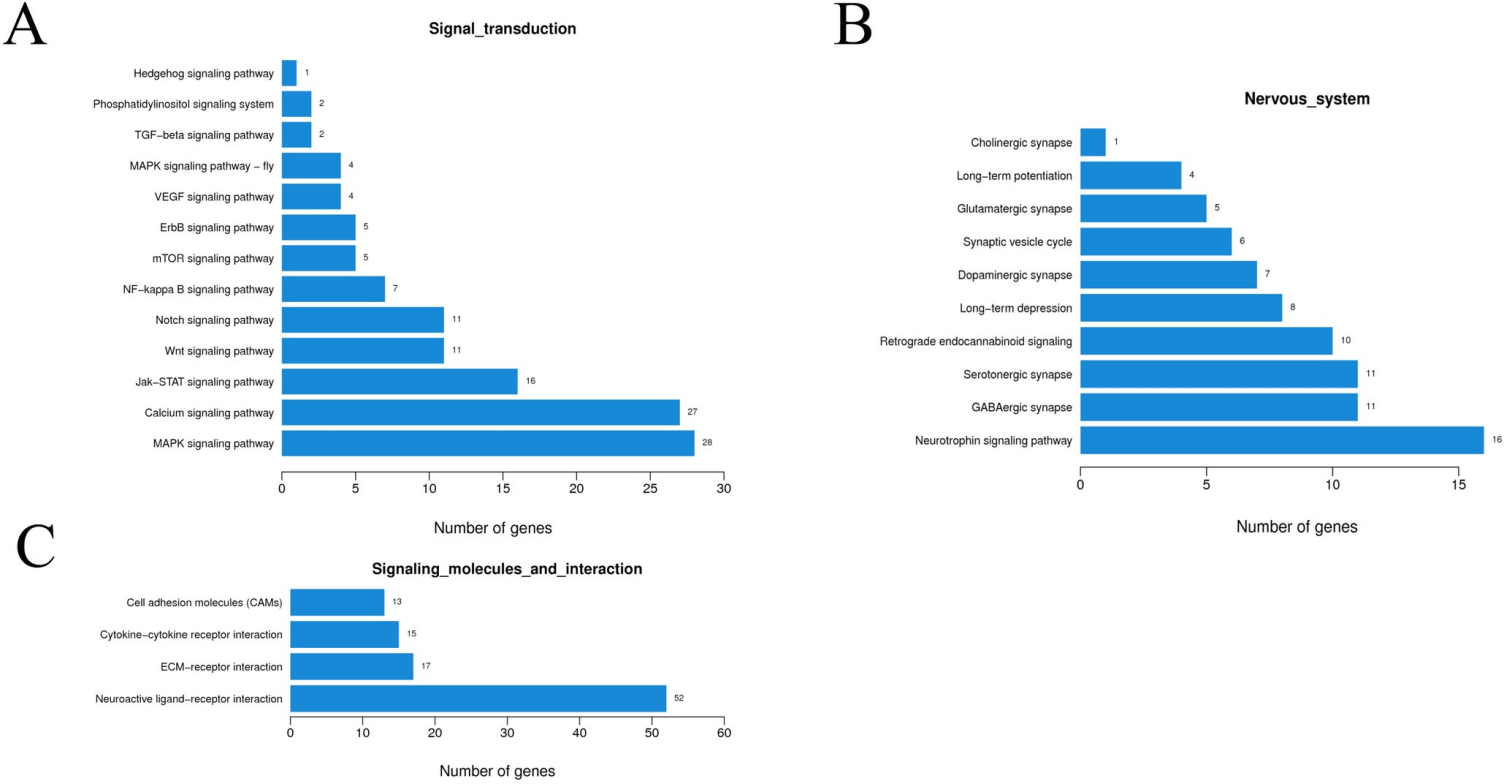
KEGG pathway enrichment of differential genes compared between knockdown insects and control at 6 h. (A) The differentially expressed genes in the signal transduction. (B) The differentially expressed genes in the signaling molecules and interaction. (C) The differentially expressed genes in the nervous system.

The KEGG pathway analysis indicated the signal transduction was enriched in the MAPK and calcium signaling pathways (Fig 4A). The signaling molecules and interaction were enriched in the neuroactive ligand-receptor interaction (Fig 4B). In view of these results, the nervous system pathways were checked. Neurotrophin was the first enriched pathway, followed by the GABAergic synapse and the serotonergic synapse (Fig 4C). Browsing the map of these signaling pathways, both 14-3-3 protein and c-Jun were up-regulated in the neurotrophin, calcium, and MAPK pathways, which might promote neuron apoptosis (S3 Fig). At the same time, both CaMK and RhoGDI were up-regulated, which might promote axonal growth. There was a contest between devastating effects and regeneration action. Obviously, the devastating effects dominate the present cells. In the other pathways, the SERT protein to carry 5-HT was down-regulated, releasing blood vessel pressure. GABA is a type of inhibitory neurotransmitter. GABAergic synapse was up-regulated in the knockdown insects. These neural changes might respond to the aconitine toxicity and the underlying mechanisms need to be studied further.

### Differential expression of genes in the active insects

**Fig 5:**
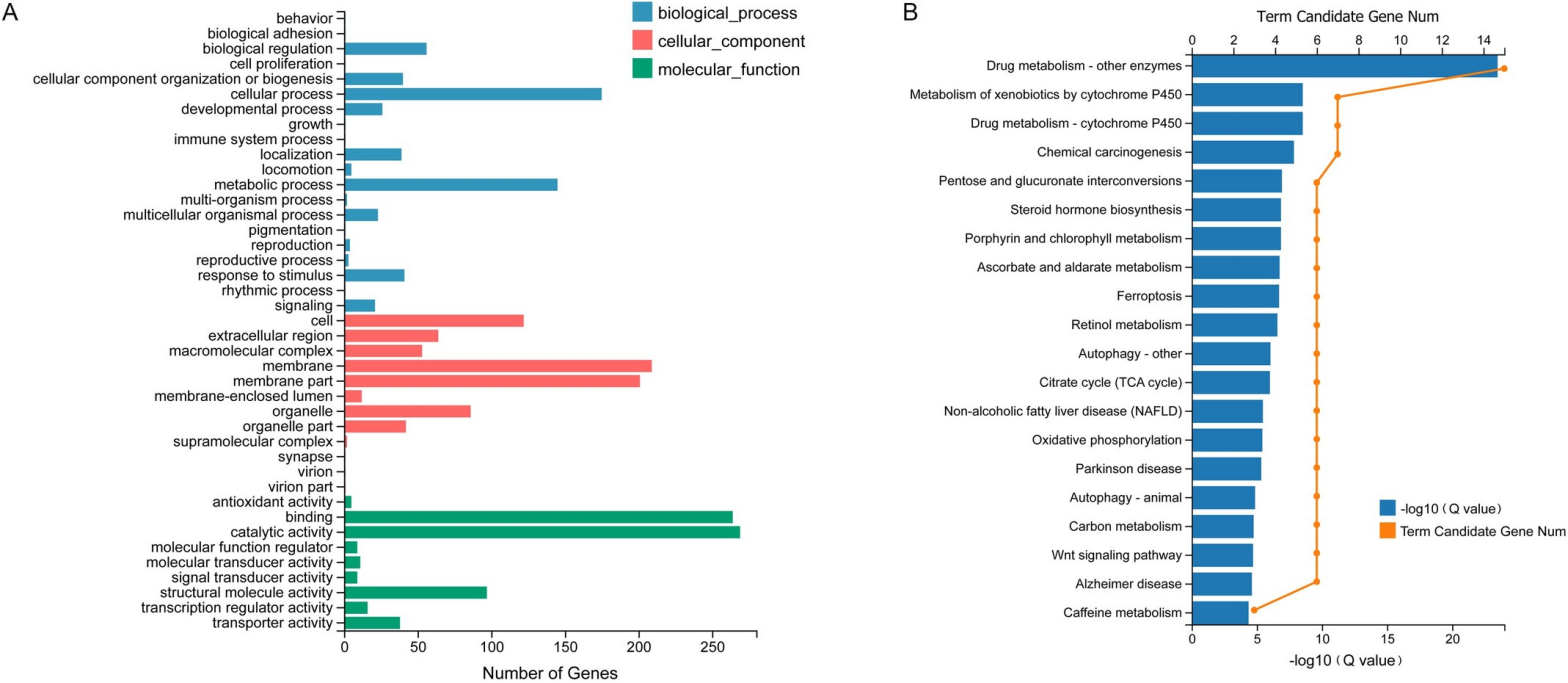
GO enrichment of differential genes compared between knockdown and active insects at 6 h. (A) GO enrichment of differentially expressed genes. (B) The differentially expressed genes in the xenobiotics biodegradation and metabolism term.

To understand the molecular biology helping the brown planthoppers to maintain activity after aconitine treatment, the differentially expressed genes between the knockdown and active insects at 6 h were analyzed. All differentially expressed genes were enriched into 41 GO classes (Fig 5A). Similarly, the xenobiotics biodegradation and metabolism pathway enriched the drug metabolism by P450s and other enzymes (Fig 5B), and UDPGT (UGT) was down-regulated (S4 Fig). It was unexpected that beta-glucuronidase (EC3.2.1.31), xanthine oxidase (EC1.17.3.2), and GMP synthase [glutamine-hydrolyzing] (EC6.3.5.2), which are involved in drug metabolism, were up-regulated in the knockdown nymphs compared with that of the active ones (S5 Fig). The results might imply that for the knockdown nymphs to be awakening required mobilization of multiple enzymes to detoxify. These results should be studied further. In the drug metabolism cytochrome P450 pathway, nicotinamide N-methyltransferase (UGT and EC2.1.1.1) and glutathione S-transferase (GST, EC2.5.1.18) were down-regulated in the knockdown nymphs, indicating that the active nymphs had an increased detoxification ability.

**Fig 6:**
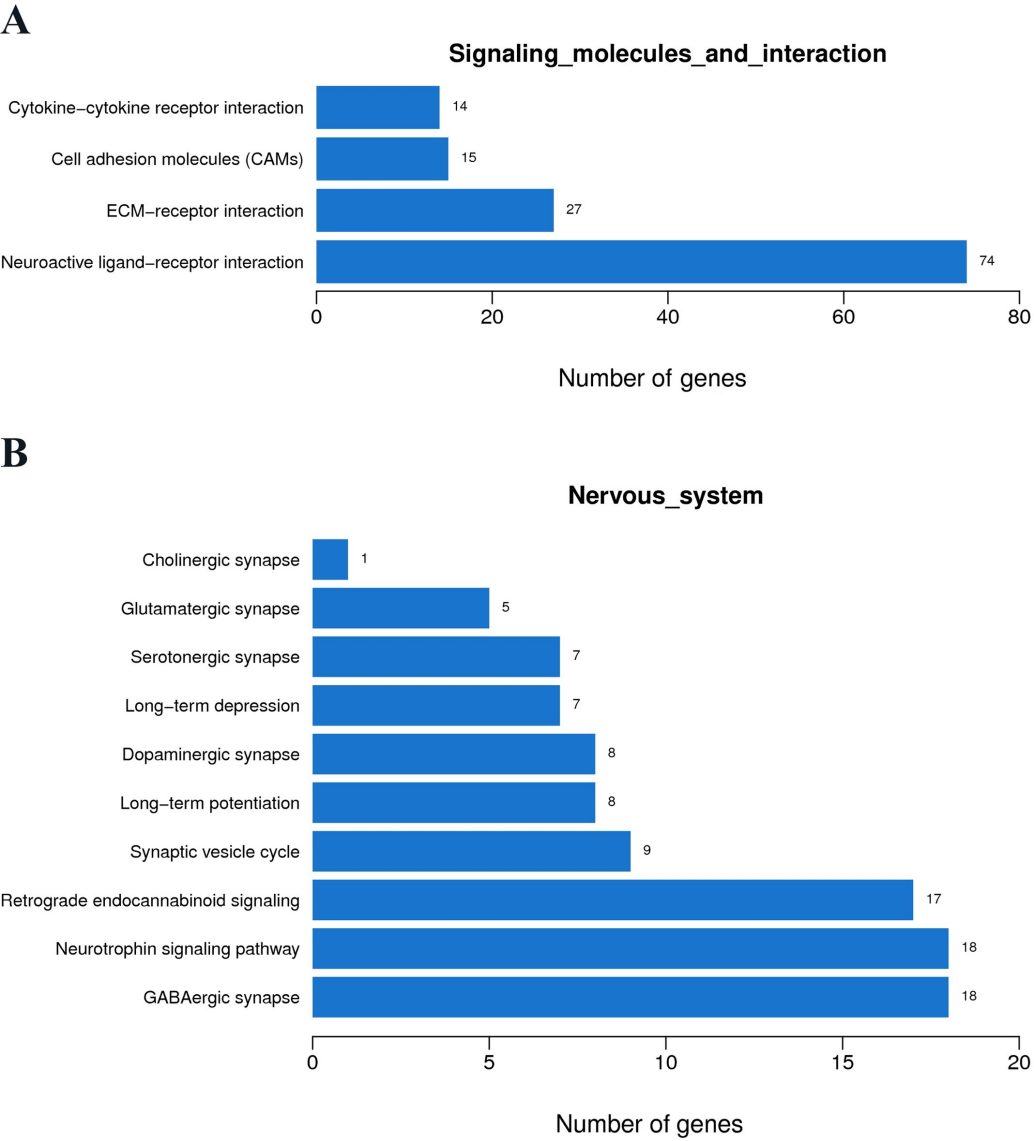
KEGG pathway enrichment of differential genes compared between knockdown and active insects at 6 h. (A) The differentially expressed genes in the signaling molecules and interaction. (B) The differentially expressed genes in the nervous system.

From the KEGG pathway analysis, the signal transduction was enriched in the MAPK signaling pathway. The signaling molecules and interaction were enriched in the neuroactive ligand-receptor interaction (Fig 6A). In view of this, the nervous system pathways were investigated (Fig 6B), where the GABAergic synapse, the neurotrophin signaling pathway, and the retrograde endocannabinoid synapse were enriched. Browsing the map of these signaling pathways, the GABA synapse function of the active insects was increased. At the same time, CaMK in the active insects was up-regulated which might promote neuron outgrowth.

## Discussion

The brown planthoppers severely damage rice production and are a challenge to crop protection scholars. Aconitine is a type of alkaloid that is produced by the *Aconitum* plant. In vertebrates, aconitine acts on the voltage-dependent sodium-ion channels and cause an anesthetic effect with diarrhea, convulsions, arrhythmias, or death. Aconitine has the potential to be an environment-friendly insecticide with its degradable composition. In the current study, the nymphs exhibited aconitine resistance to some degree. Therefore, understanding the resistance mechanism would help in developing aconitine into a novel insecticide.

As the results showed, the CYP3A4 and glucuronosyltransferase (UGT) enzymes decreased at 6 h following exposure in the knockdown nymphs compared with that of the control. The cytochrome P450-involving metabolism is a common mechanism by which insects become resistant to insecticides, as reported for various species and insecticides [17–25]. UDPGT is reported to be involved in the xenobiotics metabolism of insects and with resistance to insecticides [18, 19]. CYP3A4 is a member of cytochrome P450s and UGT is a type of UDPGT. In the current study, the down-regulation of both CYP3A4 and UGT were the mechanisms associated with the knockdown effects of aconitine.

GSTs help insects to obtain insecticide resistance in some species [18, 19, 21, 22, 25, 26]. In the current study, GSTs were up-regulated in the awake nymphs compared with those that were knockdown susceptible at 6 h. Such upregulation was associated with maintaining consciousness.

GABA receptors in the nervous systems of insects are the targets of many insecticides. The mutation of GABA receptors is associated with resistance to multiple insecticides [18, 19]. The GABA receptor-mediated aconitine resistance is a type of target site resistance. In the current study, upregulation of GABAergic synapse was observed in all comparisons, indicating the GABA receptor mutations were involved in the resistance to aconitine in the brown planthopper nymphs.

In practice, a suitable high dosage of aconitine can kill nymphs, and low dosage only knockdown them. At the same time, some brown planthopper nymphs would be resistant to aconitine naturally through the abovementioned mechanisms. Thus, aconitine toxicity has the potential to control the sensitive brown planthopper nymphs. To deal with the resistant nymphs, the insecticides of CYP3A4 inhibitor or glucuronosyltransferase inhibitor should be used together with aconitine. As a potential lead compound, aconitine showed a good insecticidal effect. Although it has toxicity to mammals, the structure of aconitine could be optimized to increase its selectivity between mammals and insect pests with further study. In addition, aconitine is fully degradable without environmental residual and would be safe for humans with the correct use (S2 File).

Compared with the transcriptomes of the knockdown and control insects, the genes potentially related to aconitine stress were identified, including the enzymes CYP3A4, glucuronosyltransferase (UGT), GST, carboxylesterase, and GABAergic synapse. The results provide useful information for uncovering the mechanism of toxicity for aconitine against insect pests. For further research, the potential action mechanism of aconitine on the brown planthopper needed be verified by traditional experiments.

## Supporting information

S1 Fig GO enrichment of differential genes compared between knockdown insects and control at 6 h. The drug metabolism pathway including P450s.
(TIF)

S2 Fig GO enrichment of differential genes compared between knockdown insects and control at 6 h. The drug metabolism pathway other enzymes.
(TIF)

S3 Fig KEGG pathway enrichment of differential genes compared between knockdown insects and control at 6 h. The differentially expressed genes in the neurotrophin signaling pathway.
(TIF)

S4 Fig GO enrichment of differential genes compared between knockdown and active insects at 6 h. The drug metabolism pathway P450s.
(TIF)

S5 Fig GO enrichment of differential genes compared between knockdown and active insects at 6 h. The drug metabolism pathway and other enzymes.
(TIF)

S1 File Toxic effect of aconitine on *Nilaparvata lugens*. (DOCX)

S2 File Detection and analysis of aconitine residue. (DOCX)

S1 Table General information of the transcriptomes. (DOCX)
