# Supplementary figures and images for: Insecticidal effect of aconitine on the rice brown planthoppers
Source: PLoS One. 2019 Aug 19;14(8):e0221090. doi: 10.1371/journal.pone.0221090 (PMC6699874; doi:10.1371/journal.pone.0221090)

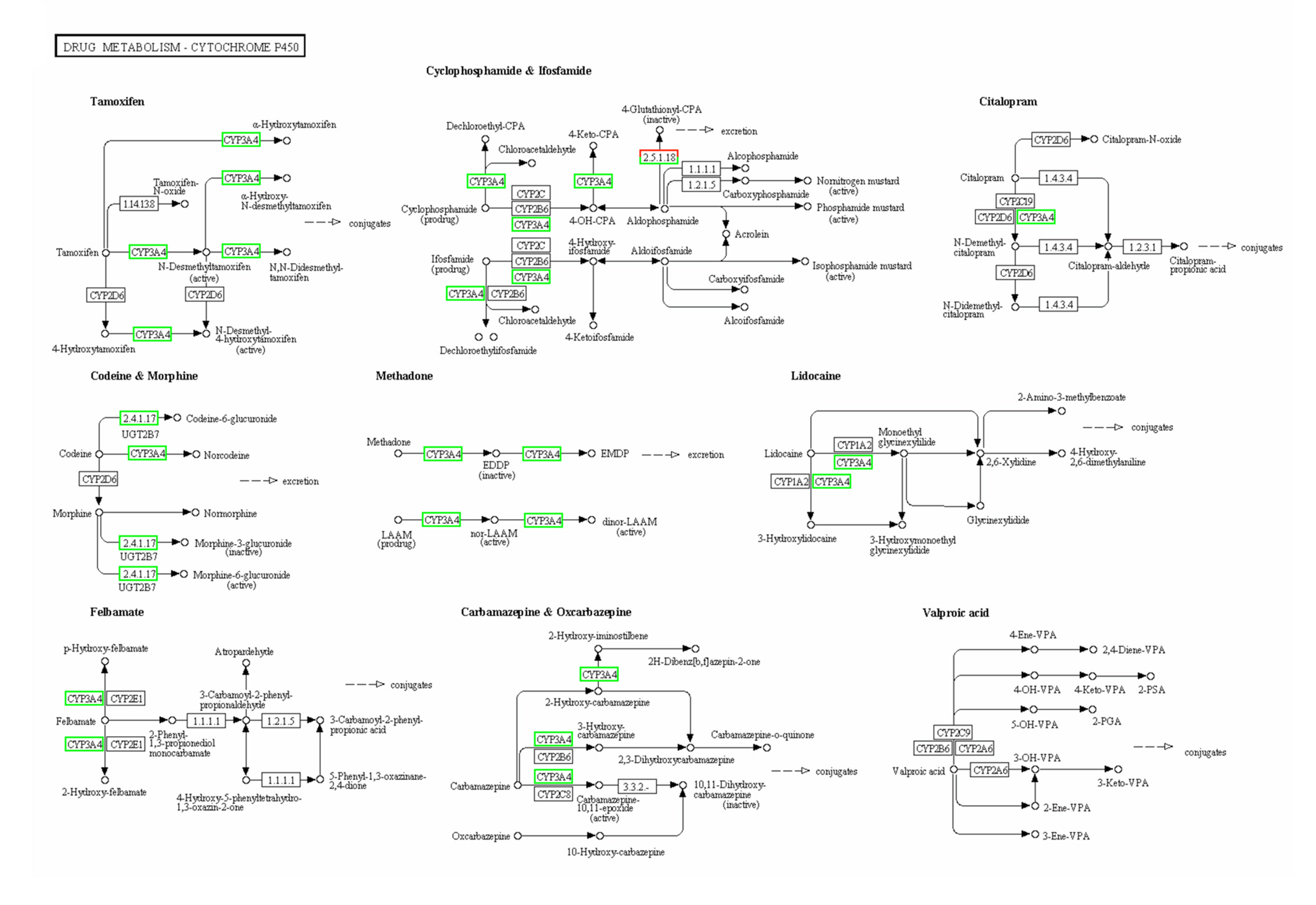

Supplement: S1 Fig — The drug metabolism pathway including P450s. (TIF) [file pone.0221090.s001.tif]

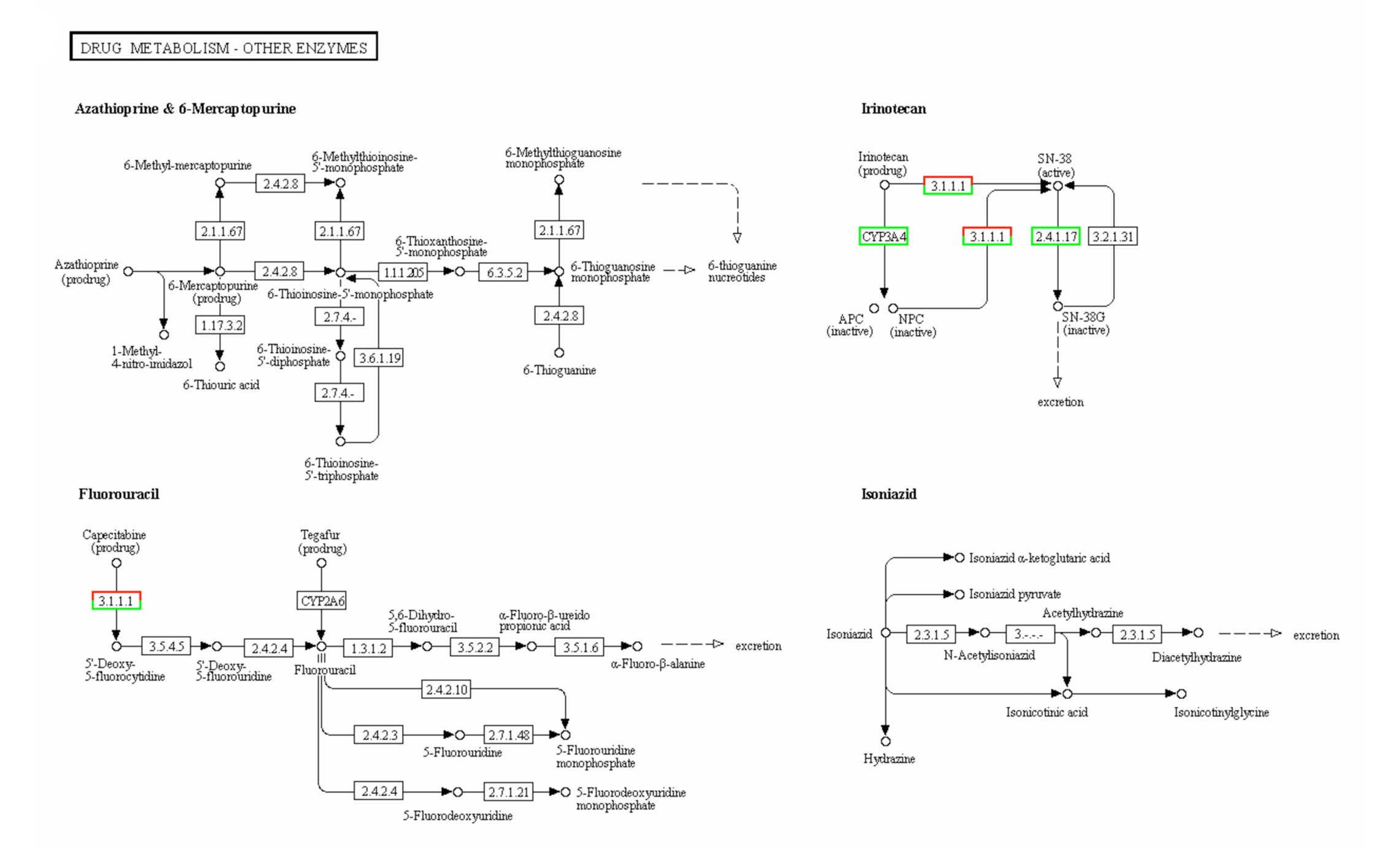

Supplement: S2 Fig — The drug metabolism pathway other enzymes. (TIF) [file pone.0221090.s002.tif]

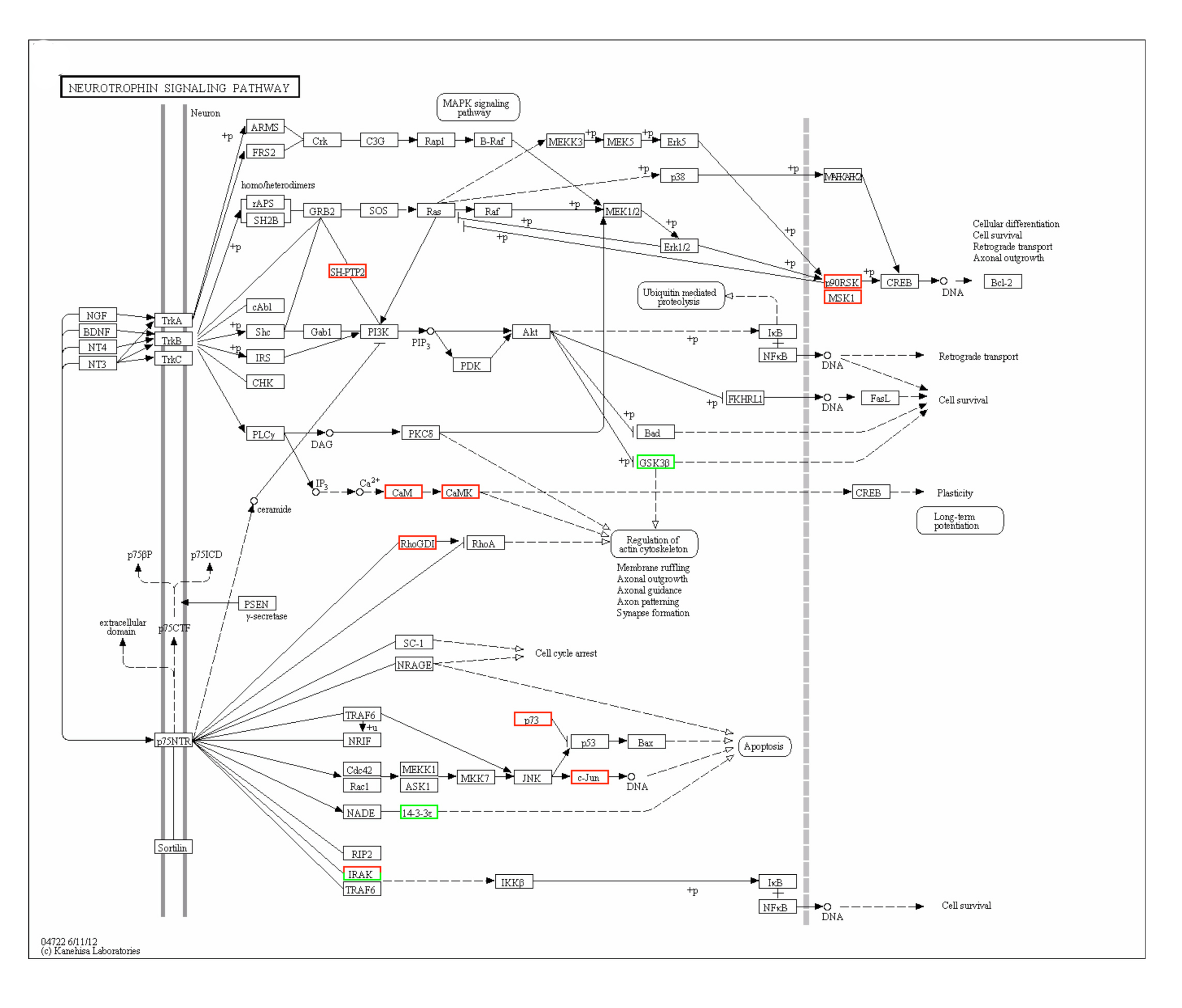

Supplement: S3 Fig — The differentially expressed genes in the neurotrophin signaling pathway. (TIF) [file pone.0221090.s003.tif]

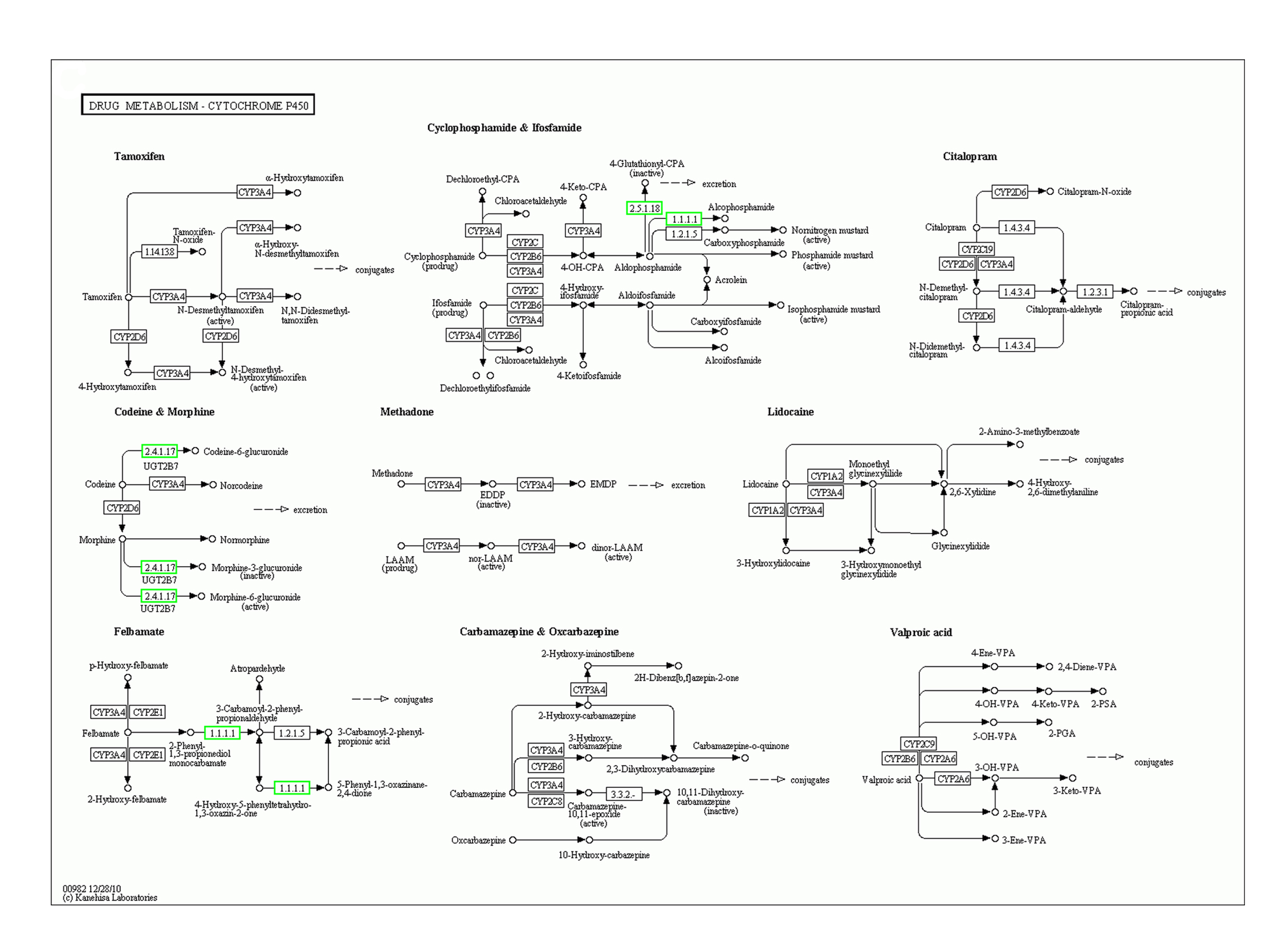

Supplement: S4 Fig — The drug metabolism pathway P450s. (TIF) [file pone.0221090.s004.tif]

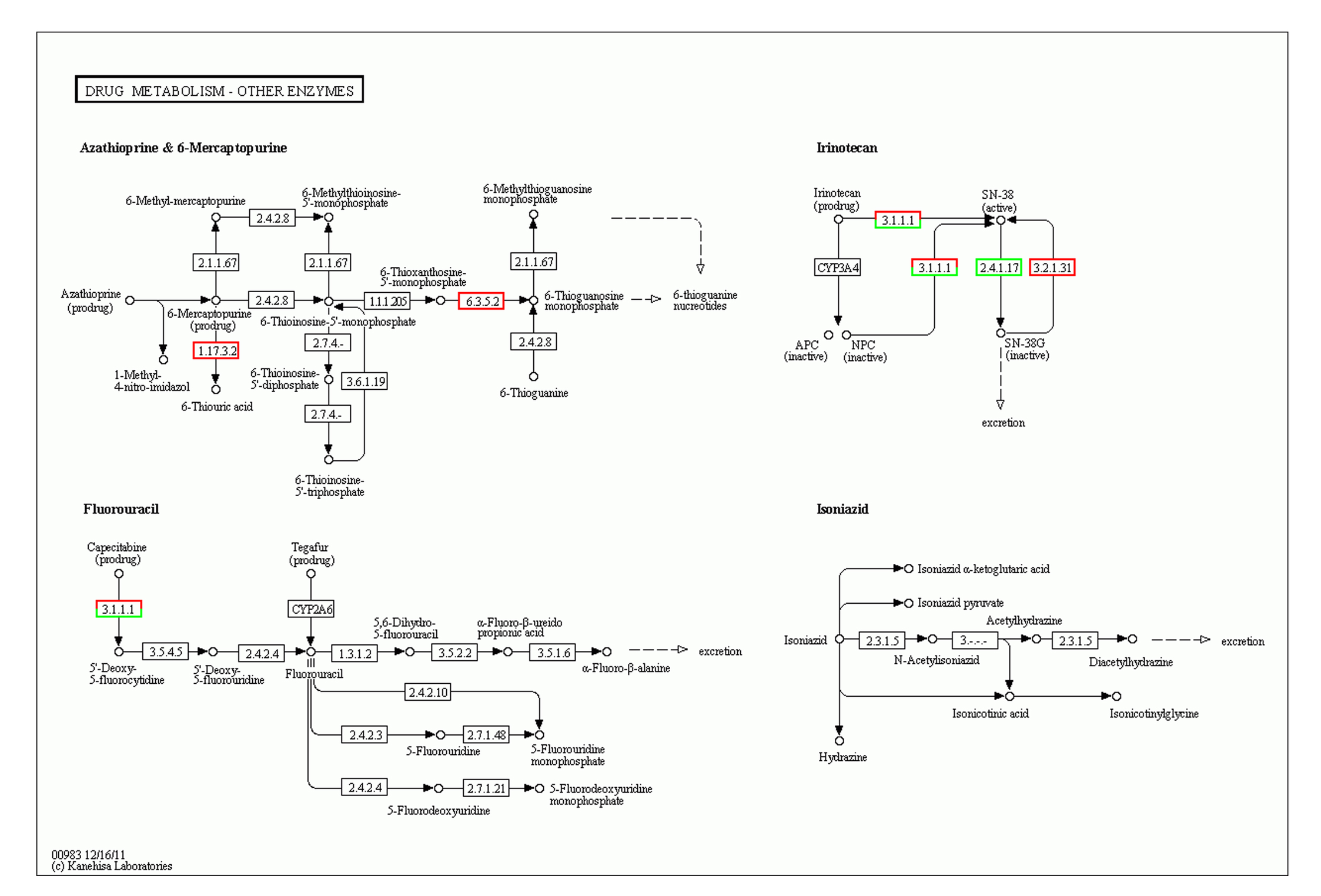

Supplement: S5 Fig — The drug metabolism pathway and other enzymes. (TIF) [file pone.0221090.s005.tif]
