# Supplementary material for: Insecticidal effect of aconitine on the rice brown planthoppers
Source: PLoS One. 2019 Aug 19;14(8):e0221090. doi: 10.1371/journal.pone.0221090 (PMC6699874; doi:10.1371/journal.pone.0221090)
Supplement: S1 File — (DOCX) [file pone.0221090.s006.docx]

**Detection and analysis of aconitine residue**

**Materials and methods**

**Materials**

Rice stems and leaves

The samples were collected from the experimental field of Jilin Agricultural Science and Technology University on June 20, 2017. 1600 mg/L aconitine was sprayed on the leaves and stems of rice, before being cut 24 hours later. The aconitine attached to the surface was washed off with ethanol and the leaves and stems of rice were dried and crushed to be used.

Rice grains

The rice seeds sprayed with aconitine were harvested on September 30, 2017 from the experimental field of Jilin Agricultural Science and Technology University, before being placed in a −80 °C ultra-low temperature refrigerator for storage.

**Methods**

**Preparation of standard solution**

An aconitine and isopropanol-trichloromethane (1:1) solution was prepared into 0.3 mg/mL. 10.0 g rice grain or stems and leaves were weighed accurately, placed into a corked conical bottle and 3.0 mL ammonia test solution and 50 mL isopropanol-ethyl acetate (1:1) added, which was weighed. The samples were extracted using an ultrasonic-assisted method of 300 W, 40 kHz, at 25 °C for 30 min. Isopropanol-ethyl acetate (1:1) solution was added to the original weight and a 25.0 mL solution was sampled and concentrated to be 3 mL at 40 °C for HPLC analysis.

**Chromatographic conditions**

A Shimadzu liquid chromatography system was equipped with a quaternary solvent delivery system, an autosampler, and a detector. A DIAMONSIL C18 column (150 mm × 4.6 mm, 5 μm) was applied for HPLC analysis. Order of processing was A: acetonitrile-tetrahydrofuran (25:15); B 0.1 mol/L ammonium acetate solution (0.5 mL of added glacial acetic acid per 1000 mL) used as mobile phase in gradient elution mode (0→46 min A: B= 15:85→26:74；46→51 min A: B= 15:85). The effluent was monitored at 235 nm and the flow rate was 1.0 mL/min, and column temperature of 40 °C.

**Sample solution detection**

The sample solution was filtered using a 0.45 μm microporous membrane, and the filtered sample placed in a bottle to detect and determine The content.

**Results and analysis**

**Chromatograph results of aconitine**

**Fig. 1 Aconitine chromatogram of Aconitine standards**

The results showed that aconitine was ideal for the peak shape of the sample as shown in Fig. 1, which met the requirements for determination of aconitine.

**Sample detection**

**Absorption of aconitine from rice stems and leaves**

**Fig. 2 HPLC chromatogram of sample**

As can be seen from Fig. 2, the sample treated with 1600 mg/L aconitine solution and in the gradient elution in HPLC, the baseline drifted slightly, due to chlorophyll and other substances in rice which has a slight impact on the detection results. However, the repeated detection results showed that there were no peaks at the retention time of aconitine (41 min) seen in the chromatogram (Fig. 2). The results showed that aconitine could not be absorbed by rice stems and leaves, so it was safe under some conditions for pesticide development.

**Detection of rice grain residue**

**Fig. 3 Chromatogram of Sample**

As can be seen from Fig. 3, there was no peak shape at 41 min from rice grains which were sprayed with 1600 mg/L aconitine solution. It can be considered there was no residue. The method was relatively safe, will not cause harm to people eating the treated rice.

**Conclusion**

The above results indicates that aconitine could not be absorbed by rice stems and leaves, and there was no residual aconitine in rice grains after harvest. Aconitine was safe for human consumption when used correctly. Therefore, although aconitine is toxic to vertebrates, poisoning can be avoided. In addition, with the development of scientific research, aconitine chemical structure modification or chemical synthesis can also be considered to reduce the toxicity of aconitine in the future. At present, this experiment was only a small experiment and was carried out under semi-closed conditions and requires further in-depth study.
