# Supplementary material for: Insecticidal effect of aconitine on the rice brown planthoppers
Source: PLoS One. 2019 Aug 19;14(8):e0221090. doi: 10.1371/journal.pone.0221090 (PMC6699874; doi:10.1371/journal.pone.0221090)
Supplement: S2 File — (DOCX) [file pone.0221090.s007.docx]

## **Toxic effect of aconitine on *Nilaparvata lugens***

**Materials and methods**

**Materials**

Aconitine with a content of 98.72% was purchased by Chengdu Manslitt Biotechnology Co., Ltd. and Chengdu Institute of Biology, Chinese Academy of Sciences. The sensitive strain of *Nilaparvata lugens* was supplied by the agricultural insect and pest control laboratory of Nanjing Agricultural University. *Nilaparvata lugens* was housed in a laboratory incubator with a temperature of 26 ± 1 °C, a relative humidity of 70–80% and light and dark cycle of 16 h/8 h.

Five rice seedlings were washed via the root with water, placed in a culture cup (top diameter 7 cm, bottom diameter 6 cm, and height of 11 cm), and the agar was poured at low temperature and allowed to set to create the test cup.

**Toxicity of aconitine to *N. lugens* by microdot drop bioassay in laboratory**

The test insect used in this experiment was *N. lugens*, a 4th instar sensitive strain of brown planthopper (brown planthopper). Aconitum was diluted with ethanol into a series of five-step concentration gradient (50,100, 200, 400, and 800 mg/L). After *N. lugens* was anaesthetized with CO_2_, a hand-held microinjector (UMP2 ultra-micro pump, Salasota, Florida, USA), was used to place a small drop (0.025 μL) of the solution onto the front chest backplate of *N. lugens*. Each concentration was used to treat 30 *N. lugens* and repeated three times, with the control group treated with ethanol instead of aconitum. After each treatment, 30 *N. lugens* were put into different measuring cup, sealed with gauze, and the test cup cultured maintaining the temperature 25 ± 1 °C and 16 h light / 8 h dark period so the mortality rate examined 48 h later. When *N. lugens* was blackened, shriveled, or immobile after being touched with a brush, it was thought to have died during the investigation.

## **Results and analysis**

**Assay of toxicity of aconitine to *N. lugens* larvae by microdot drop method**

**Table 1 Toxic effect of aconitine on the 4th instar larvae of *N. lugens* by microdrop method (48h)**

| **Concentration（mg/L）** | **Effective dose logarithm** | **Average mortality（%）** | **Adjusted mortality（%）** | **significance of difference** |
| --- | --- | --- | --- | --- |
| CK |  | 6.67±0 |  | a |
| 50 | 1.6990 | 15.56±1.92 | 9.52 | b |
| 100 | 2.0000 | 22.22±6.94 | 16.67 | c |
| 200 | 2.3010 | 27.78±7.70 | 22.62 | d |
| 400 | 2.6021 | 53.33±3.33 | 50.00 | e |
| 800 | 2.9031 | 81.11±6.94 | 79.76 | f |

**Note: the data in the table are the mean ± standard deviation of three repeats, with 30 larvae per repetition. The significant difference was found in the new repolarization test at the level of P=0.05.**

It can be seen from Table 1 that aconitine had certain contact poisoning on the 4th instar larvae of *N. lugens*, and the mortality of the 4th instar *N. lugens* increased with the increase in treatment concentration. The difference was significant between each treatment group and the control group. The toxicity regression equation y = 1.7447x 3.3415 (R^2^ = 0.9366) was obtained by linear regression between logarithm value of the effective dose and the corrected probability value of mortality, according to the dripping test results of aconitine on the larvae of *N. lugens* at the early stage of 4th instar. According to the regression equation, the median lethal dose (LD50=8.9247 ng / head) was obtained, with a 95% confidence interval of 7.4077 and 10.7523.

## **Conclusion**

The above results indicated that high concentration of aconitine has an insecticidal effect on the 4th instar larvae of *N. lugens*. The main ·method of insecticidal action was contact killing, but this does not rule out other insecticidal effects, such as refusing to eat, killing eggs, and affecting the survival of offspring larvae. The median lethal dose (LD_50_=8.9247-22.6826 ng/head) was obtained according to the regression equation of microdrip test. When aconitine reached a certain concentration, the lethality rate of brown planthopper to *N. lugens* was 100% by contact killing.
