# Supplementary material for: Insecticidal effect of aconitine on the rice brown planthoppers
Source: PLoS One. 2019 Aug 19;14(8):e0221090. doi: 10.1371/journal.pone.0221090 (PMC6699874; doi:10.1371/journal.pone.0221090)
Supplement: S1 Table — (DOCX) [file pone.0221090.s008.docx]

**S1 Table. General information of the transcriptomes.**

| Sample* | CK_6 | WH_6 | WQ_6 | CK_48 | WQ_48 |
| --- | --- | --- | --- | --- | --- |
| Total Raw Reads (M) | 49.26 | 49.26 | 49.26 | 49.26 | 49.26 |
| Total Clean Reads (M) | 47.69 | 47.73 | 47.74 | 47.67 | 47.64 |
| Total Clean Bases(Gb) | 4.29 | 4.30 | 4.30 | 4.29 | 4.29 |
| Clean Reads Q20(%) | 98.69 | 98.73 | 98.73 | 98.62 | 98.58 |
| Clean Reads Q30(%) | 93.97 | 94.1 | 94.11 | 93.73 | 93.50 |
| Clean Reads Ratio(%) | 96.81 | 96.9 | 96.92 | 96.77 | 96.71 |
| Total Mapping(%) | 73.18 | 73.99 | 73.31 | 73.07 | 73.27 |
| Uniquely Mapping(%) | 43.92 | 45.72 | 44.25 | 42.70 | 44.16 |
| Total Mapping(%) | 70.71 | 70.9 | 70.85 | 71.08 | 70.80 |
| Uniquely Mapping(%) | 54.59 | 55.11 | 55.09 | 55.19 | 55.15 |

* WH_6 - the knockdown insects at 6 h after exposure to aconitine;

WQ_6 - the active insects at 6 h after exposure to aconitine;

CK_6 - the active insects at 6 h after exposure to ethanol;

WQ_48 - the active insects at 48 h after exposure to aconitine;

CK_48 - the active insects at 48 h after exposure to ethanol
